# Prednisolone and mesenchymal stem cell preloading protect liver cell migration and mitigate extracellular matrix modification in transplanted decellularized rat liver

**DOI:** 10.1186/s13287-022-02711-8

**Published:** 2022-01-28

**Authors:** Atefeh Yaghoubi, Negar Azarpira, Saied Karbalay-Doust, Sajad Daneshi, Zahra Vojdani, Tahereh Talaei-Khozani

**Affiliations:** 1 Tissue Engineering Lab, Anatomy Department, Shiraz University of Medical Sciences, Shiraz, Iran; 2 Transplantation Research Center, Shiraz University of Medical Sciences, Shiraz, Iran; 3 Stereology and Morphometry Research Center, Shiraz University of Medical Sciences, Shiraz, Iran; 4 Anatomy Department, Shiraz medical School, Shiraz University of Medical Sciences, Shiraz, Iran

**Keywords:** Mesenchymal stem cell, Prednisolone, Liver, Decellularization

## Abstract

**Introduction:**

Regenerative medicine provides promising approaches for treating chronic liver diseases. Previous studies indicate that decellularized liver architecture is damaged by invading non-hepatic inflammatory cells. This study aimed to use anti-inflammatory and regenerative potency of bone marrow-derived mesenchymal stem cells (BM-MSC) and prednisolone for reducing fibrosis and balancing inflammatory cell migration into the decellularized liver scaffold.

**Material and method:**

The liver was decellularized by perfusing Sodium Lauryl Ether Sulfate (SLES), and nuclei depletion and extracellular matrix (ECM) retention were confirmed by DNA quantification, histochemical, and immunohistochemical assessments. Scaffolds were loaded with BM-MSCs, prednisolone, or a combination of both, implanted at the anatomical place in the rat partial hepatectomized and followed up for 2 and 4 weeks.

**Results:**

Labeled-MSCs were traced in the transplanted scaffolds; however, they did not migrate into the intact liver. Immunohistochemistry showed that the hepatoblasts, cholangiocytes, stellate, and oval cells invaded into all the scaffolds. Bile ducts were more abundant in the border of the scaffolds and intact liver. Stereological assessments showed a significant reduction in the number of lymphocytes and neutrophils in prednisolone-loaded scaffolds. The regeneration process and angiogenesis were significantly higher in the group treated with cell/prednisolone-loaded bioscaffolds. Collagen fibers were significantly reduced in the scaffolds pre-treated with cell/prednisolone, prednisolone, or BM-MSCs, compared to the control group.

**Conclusion:**

Loading prednisolone into the scaffolds can be a worthy approach to restrict inflammation after transplantation. Although pre-loading of the scaffolds with a combination of cells/prednisolone could not alleviate inflammation, it played an important role in regeneration and angiogenesis.

**Supplementary Information:**

The online version contains supplementary material available at 10.1186/s13287-022-02711-8.

## Introduction

The wound-healing response of the liver to the chronic lesions causes fibrosis and cirrhosis [1]. Both resident and migrating cells are involved in fibrogenesis, as the main liver regeneration response. Hepatic stellate cells have a pivotal role in response to liver injury. During liver regeneration, they undergo morphological and functional changes by a process called “activation” and cause liver fibrogenesis [2]. Crosstalking between hepatic stellate cells and injured hepatocytes, macrophages, lymphocytes, and endothelial cells induces fibrogenesis. On the other hand, interaction with natural killer cells leads to hepatic stellate cell death and fibrinogenesis reduction [3]. During fibrogenesis, the homeostasis caused by the ECM is destroyed and a large amount of the ECM including collagen type I and III are produced. On the other hand, matrix metalloproteinases (MMPs) degrade the ECM, and byproduct materials deposit. Cross-linking of collagen fibers is also observed [4].

Other cells that play a role in the pathogenesis of chronic liver damage are hepatic macrophages that originate from either the liver itself (Kupffer cells) or circulating monocytes. Depending on the type, macrophages can promote or reverse inflammation and fibrogenesis. Kupffer cells release some chemical mediators which cause the neutrophils to invade the liver. Neutrophils are involved in liver repair, but their long-term presence leads to the production of proteases and reactive oxygen species (ROS). Injured hepatocytes produce cytokines such as TGF-β, IL-1, CXCL10 [5]. These cytokines involve in the recruitment of leukocytes and induce fibrosis [6, 7]. Several types of macrophages including pro-inflammatory (M1), and tissue healing (M2) are present in the liver. M1 macrophages release TNF-α, IL-6, and IL-1 and induce inflammation, while M2 macrophages produce IL-10, TGF-β, PDGF, and EGF and have anti-inflammatory activity [5]. Macrophages have a dual role in liver fibrosis. They induce hepatic stellate cell survival, activation, and migration as well as the production of cytokines involved in fibrinogenesis [3]. On the other hand, a subset of the macrophages produces matrix metalloproteinase-9 and -12 that degrade fibrosis [8]. Also, dendritic cells have a role in the degradation of fibrosis [3]. Another immune cell involves in liver repair and fibrosis is the natural killer cells. They release proinflammatory cytokines that kill hepatocytes and decelerate liver repair [9]. At the same time, they also reduce fibrinogenesis by induction of apoptosis in the hepatic stellate cells [3]. Natural killer cells produce interferon-γ and kill the stellate cells to inhibit liver fibrosis [10]. Therefore, inflammatory cells have a dual function in maintaining the liver ECM hemostasis.

Although allogeneic transplantation is the standard therapy for patients with end-stage liver disease, factors such as the shortage of donor organs, the possibility of transplant rejection, high cost, and complications of the surgery limit the use of this method [11]. Bioartificial liver support systems and hepatocyte transplantation are other therapeutic options, but they also have restrictions. The artificial liver can be a temporary treatment for patients who are waiting to receive a liver transplant [12]. Although hepatocyte therapy can be an alternative for whole liver transplantation, its success depends on the type of liver disease [13], and the need for suppression of the patient's immune system [14]. In cases such as metabolic liver diseases and urea cycle defects, most of the patients who underwent hepatocyte therapy were subjected to whole liver organ transplantation later on. Hepatocyte therapy can be considered a bridge to finding appropriate liver for transplantation. Besides, 70% of the intraportal infused hepatocytes lose due to blood-mediated inflammatory reaction and clot formation. The transplanted hepatocytes were also surrounded by inflammatory cells. Anti-inflammatory intervention has been suggested to reduce hepatocyte loss [15]. Another candidate, as the remedy of liver diseases, is progenitors that are named oval cells in rodents. In cases of severe liver damage, the oval cells are activated by coordinated messaging networks controlled by growth factors, cytokines, and enzymes. However, the unusual activity of these cells is associated with pathological ulcers and the progression of liver cancer [16]. Other therapies such as platelet infusion also did not reduce liver fibrosis [17].

Decellularized liver scaffolds can be a potential solution for patients who need liver transplantation [18]. Although all cellular and nuclear materials are removed, the biological activity, vascular framework, three-dimensional structure, and mechanical integrity of the ECM are preserved during the decellularization process. The liver ECM and various growth factors provide the essential microenvironment to support cell attachment, migration, proliferation, and differentiation [19]. Rat decellularized liver implant has been reported to be recellularized successfully by different liver cell types including the hepatocytes, oval cells, stellate cells, cholangiocytes, fibroblasts, and adipocytes; however, immune cells, such as the lymphocytes, neutrophils, and macrophages are also abundant in the grafts. Immune cell infiltration in the transplanted scaffold leads to progress in the fibrogenesis process, maybe due to activating the stellate cells [20]. This leads to detrimental modification in the liver ECM architecture that hurts the liver cell reorganization. One of the ways to limit extended immune cell infiltration is the application of synthetic glucocorticoids such as prednisolone. Glucocorticoid administration can ameliorate the biochemical and histological symptoms of liver diseases, including autoimmune hepatitis, liver cirrhosis, and liver transplantation [21].

Mesenchymal stem cells (MSCs) are also a beneficial source for tissue engineering because they can suppress the immune system and are accessible [22]. They have been reported to be able to differentiate into hepatocytes [23] and promote liver regeneration [24]. MSC therapy was demonstrated to alleviate liver fibrosis in rats and humans [25], and improve liver function by producing various growth factors, immunomodulation, and preventing hepatocyte apoptosis [24, 26]. Besides, BM-MSCs reduce liver fibrosis by switching the macrophages from pro-fibrotic to restorative phenotype. Many BM-MSCs also die by apoptosis after injection to the liver. The phagocytosis of these apoptotic bodies led to fiber removal due to the expression of matrix metalloproteinase-12 [27]. The objectives of the present study were to optimize decellularized liver scaffolds using prednisolone and MSCs to balance the entrance of the immune cells into the transplanted scaffolds, reduce the fibrogenesis proceeding, and improve the cytoarchitecture reorganization.

## Materials and methods

### Liver decellularization procedure

The animal handling and treating accomplished according to the guidelines approved by the Shiraz University of Medical Sciences ethics committee (IR.SUMS.REC.1398.1210).

Whole-organ decellularization was performed on the liver of male Sprague Dawley rats weighing 200 ± 20 g purchased from the Comparative and Experimental Medicine center at Shiraz University of Medical Sciences. Animals were anesthetized using intraperitoneal administration of Ketamine 10% (100 mg/kg, Alfasan, Netherlands) and Xylazine 2% (10 mg/kg, Alfasan, Netherlands). Initially, 1 mL heparin (5000 UI/mL) was injected through the portal vein; subsequently, the portal vein branches were ligated. Then, the portal vein was intubated by a plastic polymer tube with a diameter of 2 mm, and the liver was perfused with 400 mL deionized water (DW) at the speed of 3/5 mL/min using a peristaltic pump (WT600-1F, Longer pump Co., USA). To drain blood from the liver, we cut the inferior vena cava. Then, it was perfused with 400 mL Sodium Lauryl Ether Sulfate 1% (SLES, Kimia Sanaat Ataman Co, Tehran, Iran) followed by washing with DW. To sterilize the interior part of the decellularized liver, we perfused 0.1% peracetic acid and then rinsed it with sterile phosphate-buffered saline (PBS). Finally, the acellular liver was separated and frozen until use.

### Characterization of the decellularized liver

#### DNA quantification assay

According to the manufacturer's instructions, DNA content analysis of intact and decellularized livers (*n* = 3) was executed using dsDNA Assay Kit (QIAGEN, Germany). The DNA yield (ng/μL) was quantified by spectrophotometer (the optical density (OD) at *γ* = 260 nm), using the Nano Drop® ND-1000 (Nanodrop Technologies Inc., Wilmington, DE, USA).

#### Histological assessments

Paraffin-embedded tissues were sectioned at 5 μm thickness. The intact and decellularized sections were stained with hematoxylin and eosin (H&E) and Hoechst (10 ng/mL in H_2_O) to confirm effective cell removal. Periodic acid Schiff (PAS), Alcian blue, Masson's trichrome, and Aldehyde fuchsin staining were performed to evaluate ECM components preservation including neutral carbohydrate, acidic glycosaminoglycans, collagen, and elastic fibers.

To evaluate the retention of collagen type I and IV, laminin, and fibronectin, we prepared unfixed frozen sections that were immunostained with biotinylated anti-collagen type I (1:250), anti-collagen type IV (1:500), anti-fibronectin (1:250), and anti-laminin (1:100) antibodies overnight at 4 °C (All from Abcam PLC, Cambridge, MA, USA). The color development was done by treating the samples with 200–400 μL streptavidin-HRP (1:10,000; Abcam, USA; ab7403) followed by incubating in 3,3′-Diaminobenzidine tetrahydrochloride (Sigma Aldrich, D5905). Finally, the samples were counterstained with hematoxylin.

#### Scanning electron microscopy (SEM)

Frozen decellularized scaffolds were lyophilized overnight (Christ Alpha 2–4LD-plus, Osterode am Harz, Germany) to prepare for SEM. The samples were coated with a thin layer of gold using Q150R-ES sputter coater (Quorum Technologies, UK) and imaged by a VEGA3 microscope (TESCAN, Czech Republic).

#### Determination of effective dose of prednisolone

Prednisolone was first dissolved in polyethylene glycol and, then, diluted in RPMI (Gibco) at doses 2, 1, 0.5, 0.25, and 0 mg/mL. The different dilutions of prednisolone were added to sterilized decellularized livers. To evaluate the effective dose for suppressing lymphocyte proliferation, we loaded 5 × 10^4^ Jurkat cell line in RPMI supplemented with 10% Fetal Bovine Serum (FBS), 10 U/mL penicillin, 10 μg/mL streptomycin, and 1% L-glutamate (all supplied by GIBCO) on each scaffold for 24 and 48 h, then, the cells were counted.

#### High-performance liquid chromatography

High-performance liquid chromatography (HPLC) was performed to assay the time-dependent prednisolone releasing pattern. At the end of the decellularization process, 0.5 mg/mL of prednisolone was perfused into the scaffolds. Then, we added 1 mL HPLC grade water (Merck, Germany) to the disc-like pieces of decellularized liver, and the released drug was evaluated after 30 min, 1, 3, 5, 24, and 48 h. HPLC was run using column C18 and a mobile phase containing 45% water and 55% HPLC grade acetonitrile at a speed of 1 mL per minute, and the eluted drug was detected at 254 nm. A series of known concentrations of prednisolone in polyethylene glycol was used as standard.

#### Cytotoxicity assay

Cell viability tests were performed using the HepG2 cell line (Pasteur institute, Iran). The cells were grown in low-glucose Dulbecco's modified Eagle's medium (LG-DMEM, GIBCO) with 10% FBS supplemented with 1% (V/V) penicillin–streptomycin and 1% L-glutamine. In summary, 1.0 × 10^4^ cells were loaded into the scaffolds containing prednisolone. To simulate the 2D condition, we seeded the HepG2 cells on the jellified alginate pre-coated plate containing the same amount of prednisolone. After 1, 3, and 7 days, the fresh media were replaced with -(4,5-dimethyl thiazolyl-2)-2,5-diphenyltetrazolium bromide (MTT, M5655; Sigma-Aldrich) at a final concentration of 0.5 mg/mL for 3 h; finally, crystalline formazan was eluted by 100 µL of 100% Dimethyl sulfoxide. Then, the absorbance was evaluated by the BMG Spectro Nano Elizabeth Reader at 595 wavelengths.

#### Mesenchymal stem cell isolation

To collect rat BM-MSCs, we inserted a 23-gauge syringe into the tibia and femur cavities in the aseptic condition and flashed it with serum-free DMEM supplemented with 10 U/mL penicillin. After centrifuging at 1200 rpm, the cell pellets were resuspended and seeded in DMEM containing 10% FBS, 10 U/mL penicillin, 10 μg/mL streptomycin, and 1% L-glutamate (all Gibco). Finally, the suspended cells were removed for 7 days, and the attached cells were allowed to grow up to confluency.

The isolated cells were characterized by flow cytometry. Briefly, permeabilization was performed by incubating 5 × 10^5^ cells with PBS containing goat serum (Gibco, USA) and Tween 20 (Merck, USA). Then, one µL of FITC-labeled anti-CD44 and -CD45, PerCP-conjugated CD90, and PE-conjugated CD34 antibodies (all from Abcam) was added. After washing with PBS (1–2 mL), the samples were centrifuged at 1200 rpm, fixed by 1% paraformaldehyde, and assessed by a 4-color FACScalibur flow cytometer (BD FACSCaliburTM; BD Biosciences) along with CellQuest Pro software. The data were analyzed by flowjo software.

The BM-MSCs were also exposed to the osteogenic (Gibco) and adipogenic media (Gibco) for four and three weeks, respectively. Then, the differentiated cells were fixed with 4% paraformaldehyde and stained with Alizarin Red S (Sigma, USA) and Oil Red O (Sigma, USA) to detect calcium resorption and oil droplets, respectively.

#### Attachment test

To evaluate the possible impact of prednisolone loading on cell adhesion and infiltration, we seeded MSCs at a density of 2 × 10^5^/mL on the prednisolone-free or loaded scaffolds and incubated them, as described above. After 30 min, the unattached cells in the culture media were collected and counted. The number of attached cells was calculated by subtracting the unattached cells from the initial cell count.

#### Tracing of the mesenchymal stem cells

To trace the cells, we labeled them with a PKH26 red fluorescent cell linker kit (Sigma); then, they were loaded into the scaffolds by injecting 1 × 10^6^ cell/mL via the framework of the vessels. At the beginning and on the 14th day after transplantation, the scaffolds were freshly cryosectioned and counterstained by Hoechst, and observed by fluorescent microscopy.

#### Experimental design and liver scaffold transplantation

**Fig. 1:**
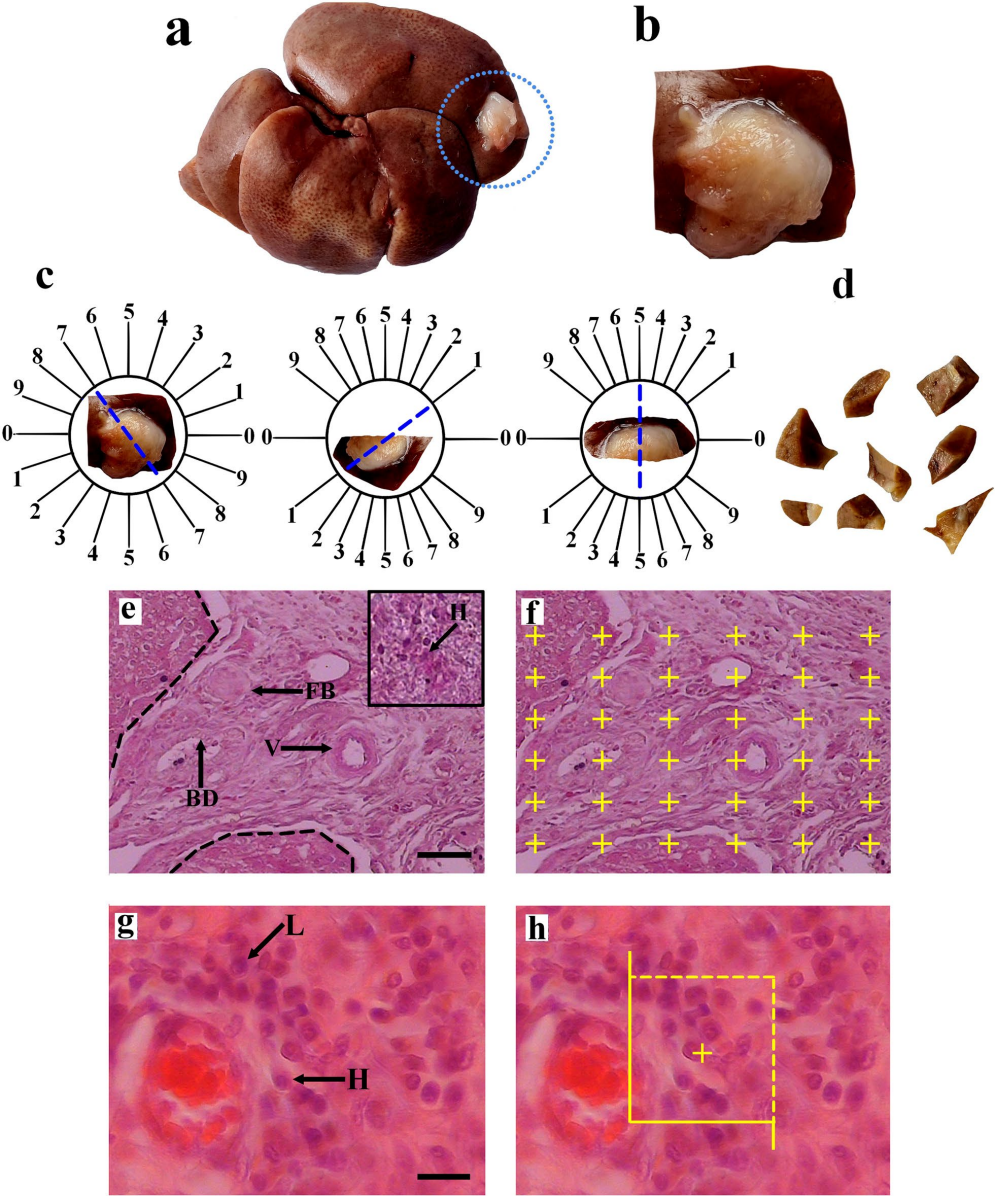
The whole liver and implant (**a**), and higher magnification of the transplanted decellularized graft (**b**). Uniform isotropic random sections were obtained from the graft tissue by the orientator method (**c**). A collection of isotropic uniform random sections was obtained from the graft tissue (**d**). The borders of the graft with the normal liver are indicated on the histological section by dot line and tissue regions were characterized and in this figure, hepatocyte (H), bile duct (BD), blood vessel (V), and foreign body (FB) are seen (**e**). The point-counting method was used to estimate the volume graft components (**f**). The cells of the graft tissue were identified (**g**). The optical disector technique was used to estimate the number of cells in the graft tissue. In this figure, the hepatocytes (H) and lymphocytes (L) are seen (h). Scale bars = 30 µm in (**e**, **f**) and 20 µm in (**g**, **h**)

To evaluate the in vivo recellularization efficiency of the scaffolds, we randomly divided 40 mature male Sprague–Dawley rats weighing about 230 ± 10 g into four groups; each one received either drug-free, prednisolone-, cell-, or cell/prednisolone-loaded decellularized scaffolds. Each group was divided into two subgroups kept for either 2 or 4 weeks (Additional file 1: Fig. S1). The rats were then anesthetized by intramuscular injection of 10 mg/kg xylazine and 100 mg/kg ketamine, and a small incision below the xiphoid process was made. Using a surgical trocar, we made a cavity with a diameter of 1 cm in the left lateral lobe of the liver. Then, the same size of sterile acellular liver was implanted in the cavity. The animals were kept under the standard condition and ad libitum for two and four weeks. Finally, the grafts and surrounding intact liver (Fig. 1a, b) were removed through a midline laparotomy, and fixed in 4% formaldehyde for further analyses.

### Stereological study

#### Graft tissue preparation

Estimation of some of the stereological variables such as microvessels length needs isotropic uniform random sections; thus, the graft was cut according to the “orientator method” (Fig. 1c, d) [28, 29]. Then, the paraffin-embedding blocks were sectioned at 5 and 25 μm-thick and stained with Periodic Acid Schiff (PAS), Masson Trichrome, H&E.

A video microscopy system was employed, and the borders of the graft with normal liver tissue as well as the boundaries between the regenerated liver and inflammatory tissue regions were characterized in each section (Fig. 1e).

The inflammatory tissue regions are occupied by inflammatory cells such as the lymphocytes, neutrophils, macrophages, and foreign body giant cells; also, the regenerated liver tissue includes the hepatocytes, fibrocytes, bile ducts, vessels, and fibrous tissue.

#### Estimating the volume density of the graft components

The graft sections with a thickness of 5 µm were used to estimate the volume density of the regenerated liver and inflammatory tissue structures. The stereological tools consisted of a Nikon E-200 microscope (Nikon, Japan) joined to a computer. The probes of stereology at the final magnification of 780 X were employed in the live pictures, and the volumes of the favorite structures were evaluated by the “point-counting technique”(Fig. 1f) [28, 29]. The volume density “Vv (favorite structure/graft)” was estimated using the following formula:

$${\text{Vv}} = \sum {\text{P }}\left( {\text{favorite structure}} \right)/\sum {\text{P }}\left( {{\text{graft}}} \right)$$

where “∑*P* (structure)” and “∑*P* (graft)” were the number of points hitting the favorite structure and the graft section, respectively [28, 29].

#### Estimating the numerical density of the graft cells

The optical disector technique was employed to estimate the number of lymphocytes and neutrophils in the thick graft section (Fig. 1g). The optical disector consists of different parts, including an Eclipse microscope (E200, Nikon, Tokyo, Japan) with a high numerical aperture (NA = 1.30) × 40 oil-immersion objective, joined to a video camera, which transmits the live microscopic picture to a computer monitor, and an electronic microcator with digital readout (MT12, Heidenheimer, Traunreut, Germany) for measuring the movements in the Z-direction with an exactness of 0.5 μm. The location of microscopic fields was chosen by moving the stage in *x*- and *y*-directions at equal distances with systematic uniform random sampling.

The counting frame is a stereological probe with a stereology software system (Stereo lite, SUMS, Shiraz, Iran). The unbiased counting frame consists of two solid forbidden lines (the left and inferior borders and their extensions) and two dashed acceptance lines (the right and superior borders) that were overlaid on the live image graft at a final magnification of 1520X (Fig. 1h). The guard area is the region at the top and bottom of the graft sections. These areas were employed to avoid cutting tissue artifacts that occur throughout tissue processing on these areas of the graft sections. Any cell event in focus within the up (the first 5 μm) or down guard areas was not counted. The distance between the upper and lower guard areas was the “height of disector” which was 15 μm here.

The cell nucleus which appeared in the maximal focus within the frame, was placed completely or partially inside the counting frame and did not contact the forbidden line was chosen (Fig. 1h). The numerical density (NV) was estimated by the following formula:

$${\text{Nv}}\left( {{\text{cells}}/{\text{graft}}} \right) = \Sigma Q{-}/\left( {\Sigma p \times \left( {a/f} \right) \times h} \right) \times \left( {t/{\text{BA}}} \right)$$

where “Σ*Q*–” was the number of the cell nuclei of the graft coming into maximal focus in the disector height, “Σ*P*” was the total of the counting frames in all microscopic fields, and “*h*” was the height dissector. “*a/f*” was the area of the frame, “*t*” was the real section thickness measured with the microcator, and “BA” was the block advance of the microtome set. The thickness of the graft section was measured in the whole microscopic fields of view with uniform random sampling from each graft section[28, 29].

#### Histological and immunohistochemical evaluation after implantation

Heat-induced epitope retrieval in citrate and Tris–EDTA buffers were performed in paraffin-embedded sections. Then, the sections were stained by pre-diluted anti-c-kit, anti-cytokeratin 19 (CK19), and anti-glial fibrillary acidic protein (GFAP) primary antibodies (all from Dako, Denmark) followed by incubating in polymer-HRP and then, in diaminobenzidine. The distribution of the bile ducts was evaluated by the Voronoi tessellation method in PAS staining samples, using ImageJ software (http://mac.softpedia.com/get/Graphics/ImageJ.shtml).

### Statistical analyses

The data were presented as mean ± standard deviation, and statistical analyses were performed using two-way analysis of variance (ANOVA) analysis, Tukey, and Mann–Whitney U tests. Graph Pad Prism 9 was used for analyses. A *P* value less than 0.05 was considered significant.

## Results

### Scaffold preparation and characterization

**Fig. 2:**
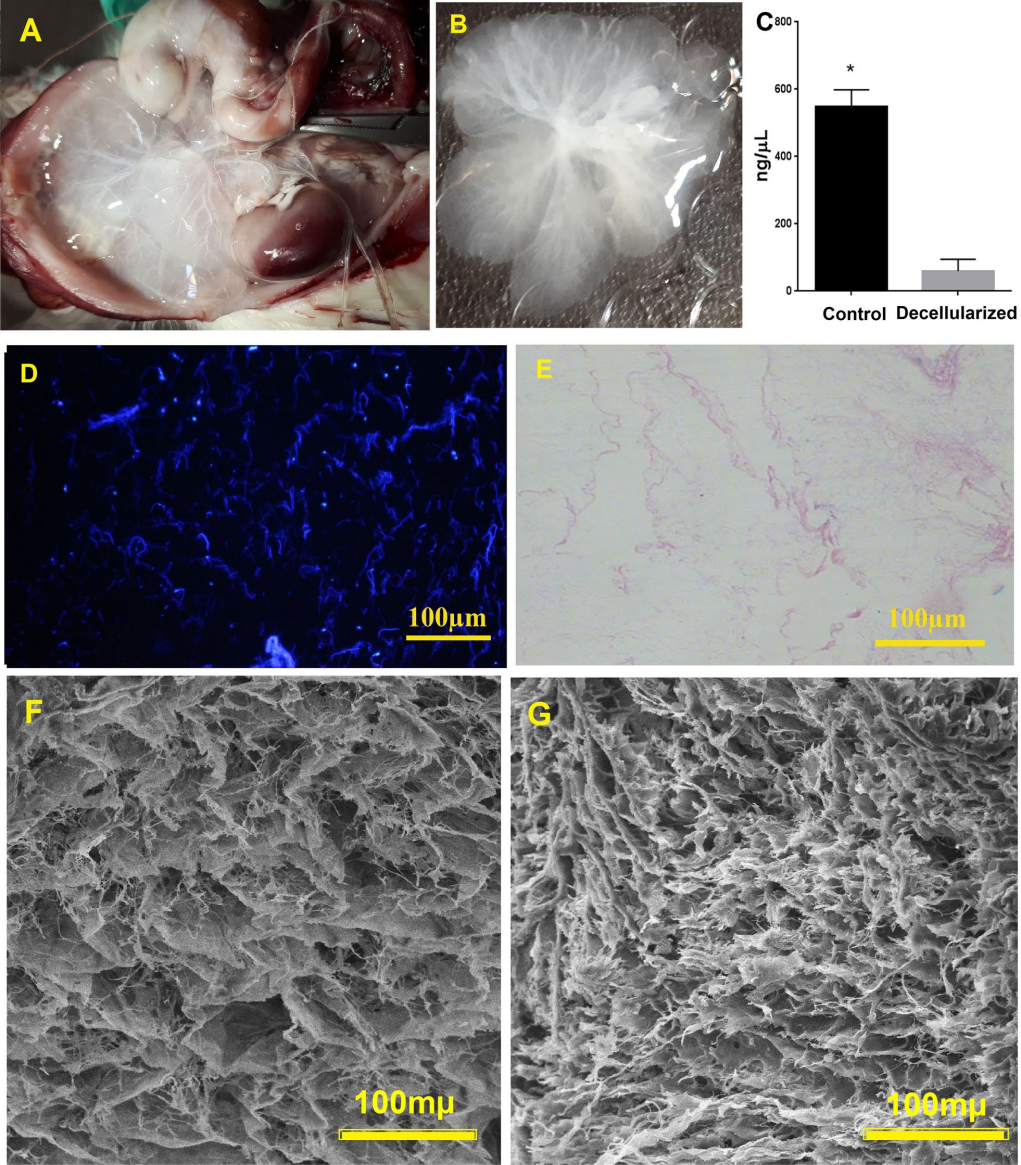
Chronological macroscopic (**A**) and microscopic changes (**B**) of the rat liver during the SLES-based decellularization process. The change of liver color from red to transparent during the decellularization procedure can be observed. DNA quantification (**C**) showed a significant reduction in the DNA content after decellularization (data are expressed as the mean ± standard error of the mean (SEM), *N* = 3 per group, *Indicates significant difference, (*P* = 0.0002). Hoechst (**D**), Hematoxylin and eosin (**E**) staining of decellularized liver confirms nuclei depletion. Scanning electron microscopy of the cut of edge (**F**) and surface (**G**) of decellularized scaffolds shows that the 3D ultrastructure remains intact

Gross observations showed that the perfused livers had a transparent appearance and visible vessel architecture (Fig. 2A, B). DNA quantification test confirmed significant DNA depletion (*P* = 0.0002, Fig. 2C), and H&E and Hoechst staining showed that the cell debris and nuclei were properly removed (Fig. 2D, E). SEM images revealed that 3D liver ultrastructure with porous appearance and the framework of the vasculature retained properly after the decellularization process. The framework of different liver parts, including hepatic lobules and portal spaces, was identified as a network of collagen fibers that provided a honeycomb appearance (Fig. 2F, G).

**Fig. 3:**
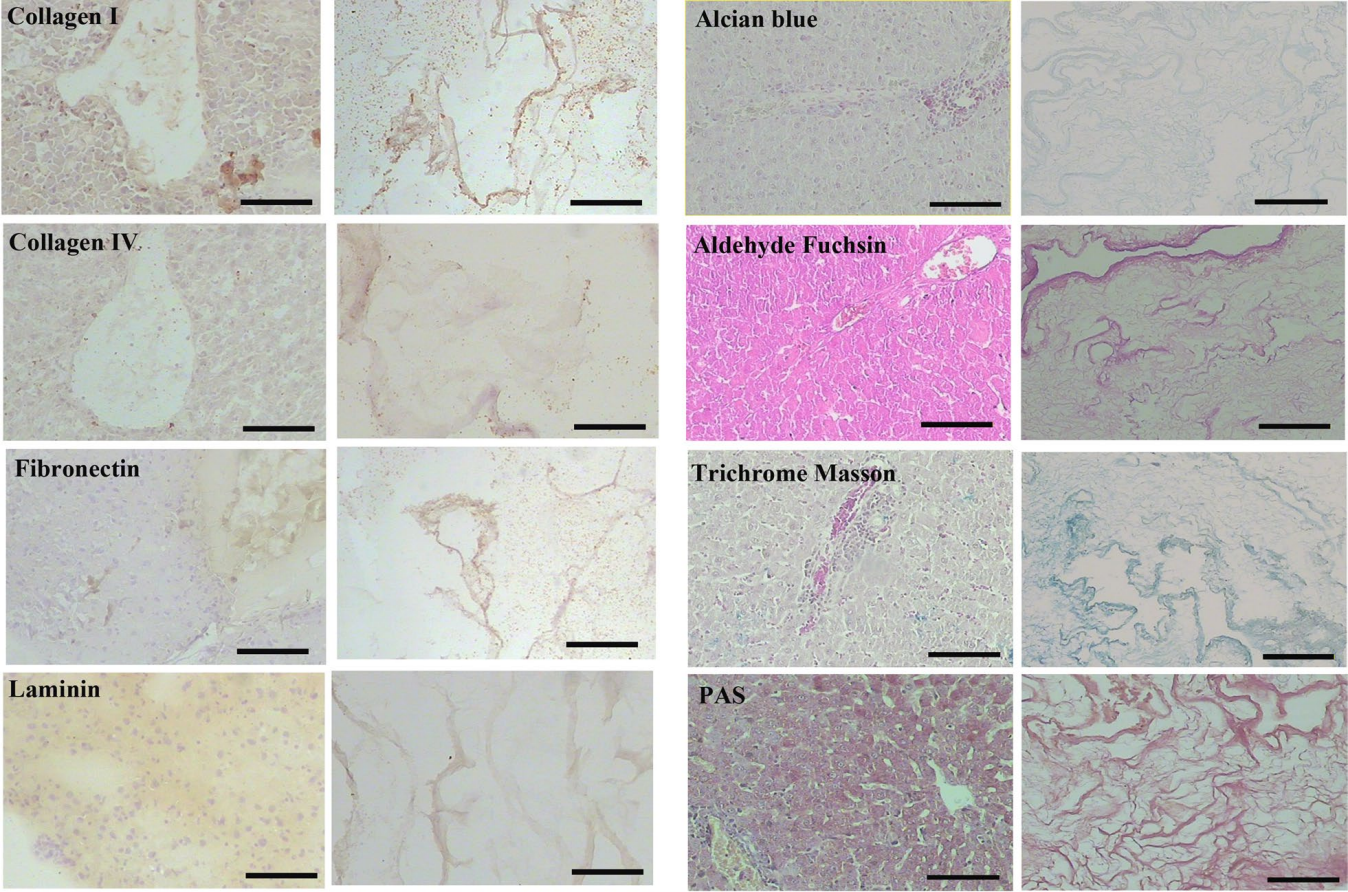
Composition preservation of the liver extracellular matrix. Alcian blue, Aldehyde-fuchsin, Masson Trichrome, and PAS for detection of the acidic glycosaminoglycans and elastic fibers, collagen fibers, and neutral carbohydrate content, respectively, revealed some extent of ECM preservation in the decellularized liver compared to the intact one. Immunohistochemistry for collagen I, IV, fibronectin, and laminin was detected to retain in the liver scaffolds with a similar distribution pattern in the intact tissue. The scale bar is 100 µm

Alcian blue, PAS, Aldehyde fuchsin, and Masson’s trichrome staining confirmed acceptable retention of glycosaminoglycans, neutral carbohydrate content, elastic and collagen fibers in the decellularized ECM compared to native liver tissue, respectively. Besides, immunohistochemistry indicated that ECM proteins such as collagen type I and IV, fibronectin, and laminin were appropriately preserved compared to the intact liver (Fig. 3).

### Dose selection

**Fig. 4:**
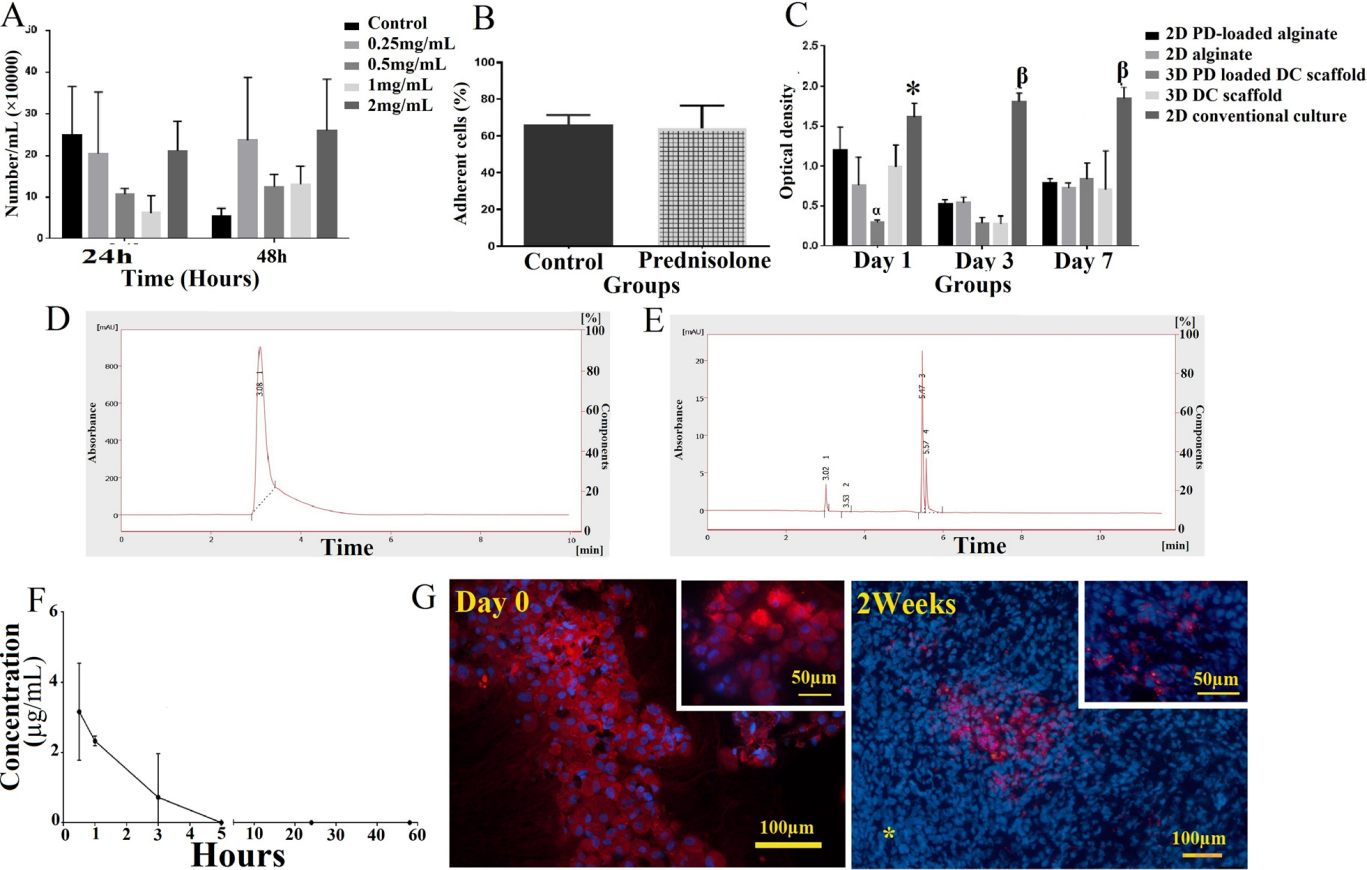
The effective dose of prednisolone was selected by exposing T-cells to a panel of doses for 48 h (**A**). Cell attachment assessments showed that prednisolone loading did not significantly affect the adhesion and penetration properties of the scaffolds (**B**). HepG2 cell line was cultured to evaluate the cytotoxicity of prednisolone-loaded scaffolds. The proliferation rate of the cells cultured in conventional 2D conditions was significantly higher than all the other groups. On the 7th day, an increase in cell viability was observed in all groups. Prednisolone showed some extent of cytotoxicity for HepG2 cell line (**C**). The drug-releasing pattern was evaluated by assessment of the released prednisolone concentrations in the medium as time progressed. Representative samples in 2 (**D**) and 5 h (**E**). The releasing curve of prednisolone from scaffolds during 48 h (**F**). Tracing of MSCs tagged by PKH26 (**G**). The injected cells were distributed uniformly throughout the scaffold at 0 day. After 2 weeks, most of the labeled cells formed large aggregates; however, some of them scattered throughout the implanted scaffold. Between the labeled cell aggregates, the untagged cells are also observed. The tagged cells were not present in the intact part of the liver (*). PD, prednisolone; DC, decellularized. *Significant difference with 2D alginate (*P* = 0.0002), and 3D PD-loaded decellularized (*P* = 0.0001) at day 1; *α* significant difference with 2D PD-loaded (*P* = 0.0001), and 3D decellularized (*P* = 0.0029) at day 1; and, *β* significant difference with all the other groups at day 3 and 7 (*P* = 0.0001)

To select the effective dose of the prednisolone, we followed up the T-cells proliferation to 48 h at the presence of a panel of various prednisolone concentrations. The low solubility of prednisolone led to sedimentation of the drug at a high concentration (2 mg/mL). Although T-lymphocyte proliferation did not significantly reduce after drug treatment, the non-significant lowest cell count was found in those treated with 0.5 and 1 mg/mL. Therefore, we finally chose 0.5 mg/mL of the drug for further tests because of both reductions of cell proliferation rate and appropriate solubility (Fig. 4A).

### Cytotoxicity, cell infiltration, and attachment assessments

On day one, MTT assessments revealed that the viable cell number was significantly lower in both 3D prednisolone-loaded (*P* < 0.0001) or prednisolone-free conditions (*P* = 0.0083) compared to conventional 2D conditions. Also, HepG2 cultured on an uncoated culture dish showed a significantly higher cell proliferation than the cells cultured on prednisolone-free alginate (*P* = 0.0002). The significantly lowest viable cells were found on 3D prednisolone-loaded scaffolds. Since the cells in conventional culture conditions continued proliferating up to days 3 and 7, the cell viability of the other groups remained constant and statistically the same in all other conditions. The reduction in cell viability was also observed on day 3 compared to day 7 due to the release of all the prednisolone content in the first 24 h. However, on the 7th day, cell proliferation was observed in all groups (Fig. 4B).

Statistical analyses showed that prednisolone loading did not significantly affect the adhesion and penetration properties of the scaffolds (Fig. 4C).

### Assessment of drug-releasing

The drug-releasing pattern revealed that prednisolone was present in the medium in 2 to 4 min, meaning the high level of the drug was released at the beginning of the drug-loaded scaffold incubation. The drug's releasing rate decreased over time, which may be due to the presence of a low level of the remaining drug in the scaffold. After 5 h, it reached zero indicating the complete release of prednisolone (Fig. 4D).

### Tracing of the mesenchymal stem cells

BM-MSCs were characterized by their fibroblast-like phenotype, the expression of CD90 (100%) and CD44 (99.8%) markers, and negative reaction for hematopoietic markers, CD34 (0.329%), and CD45 (2.95%). The pluripotency was also confirmed by differentiating the MSCs to adipocytes and osteoblast as well (Additional file 2: Fig. S2).

Tracing of PKH26 tagged-MSCs showed that injecting cells were distributed uniformly throughout the scaffold at 0 day of injection. After 2 weeks, the large aggregates of the labeled cells were still observed in the implanted scaffolds; however, some scattered tagged cells could also be observed. Untagged cells were also present throughout the implanted scaffolds that indicated invading the host cells into the grafts. No tagged cell migrated into the neighboring intact liver tissue (Fig. 4E).

### In vivo assessment

Gross examination showed that the transplanted scaffolds had no detrimental effect on the integrity and appearance of the intact part of the liver. No infection or adhesion was observed in the graft site, peritoneum, and abdominal organs. Also, graft rejection and animal mortality were not observed.

**Fig. 5:**
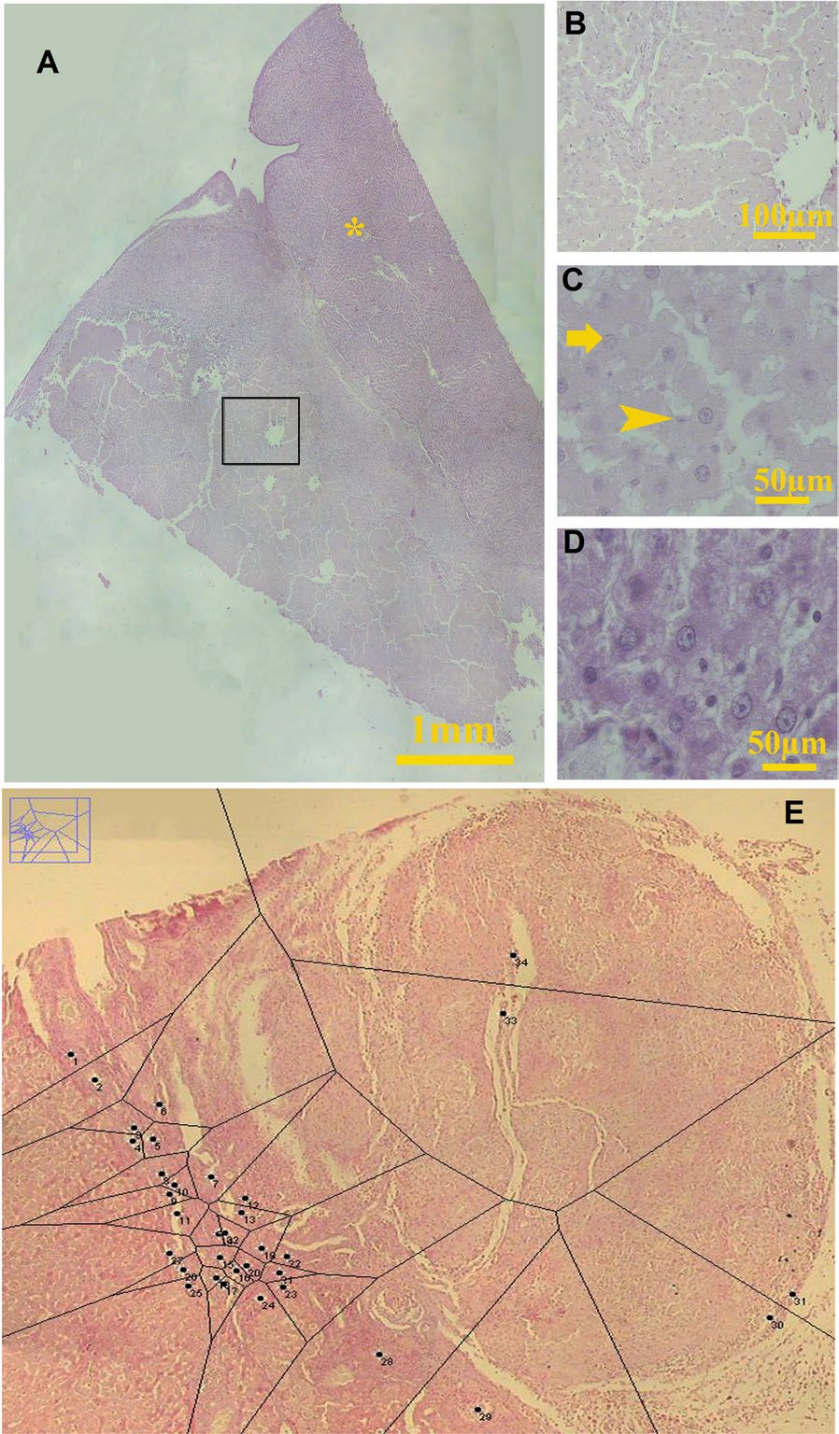
The PAS staining of the representative sample of the BM-MSC-loaded transplant showed reorganization of the hepatocyte-like cells in the form of hepatic cords (**A**). * indicates the intact part of the liver. The square in Fig **A** was examined with higher magnification and showed the reorganized hepatic cords (**B**). The higher magnification of this part (**C**) revealed the cells with hepatocyte phenotype (arrow) were arranged in the form of hepatic cords and the sinosuides could be observed between them. Arrowhead shows the nuclei of the endothelial cells. Comparing the PAS reaction of the hepatocytes in the intact part of the liver (**D**) and migrating hepatocyte-like cells in the scaffold (**C**) revealed that they had not stored glycogen yet; therefore, they were not functional. The distribution of the bile duct by tessellation method (**E**)

Microscopic studies showed extensive cell migration, and hepatocyte-like cells, fibroblasts, macrophages, lymphocytes, and neutrophils invaded into all the scaffolds in both points of time. Regardless of the type of scaffold, angiogenesis occurred and all kinds of vessels including sinusoids. In both cell-loaded scaffolds, especially those that received cell/prednisolone, the migrating hepatocytes were organized in the form of the hepatic cords (Fig. 5A, B), and the sinusoids penetrated between them (Fig. 5C). The hepatocytes were weakly stained with PAS that indicating they were immature (Fig. 5C).

In all the transplanted scaffolds, some regions resembled portal spaces containing several bile ducts, and vessels were also formed in the grafts. However, tessellation of the scanned implants revealed that the bile duct distribution was not uniform and most of the bile ducts were formed at the border of the graft and intact liver (Fig. 5D).

### Immunohistochemical evaluation after implantation

**Fig. 6:**
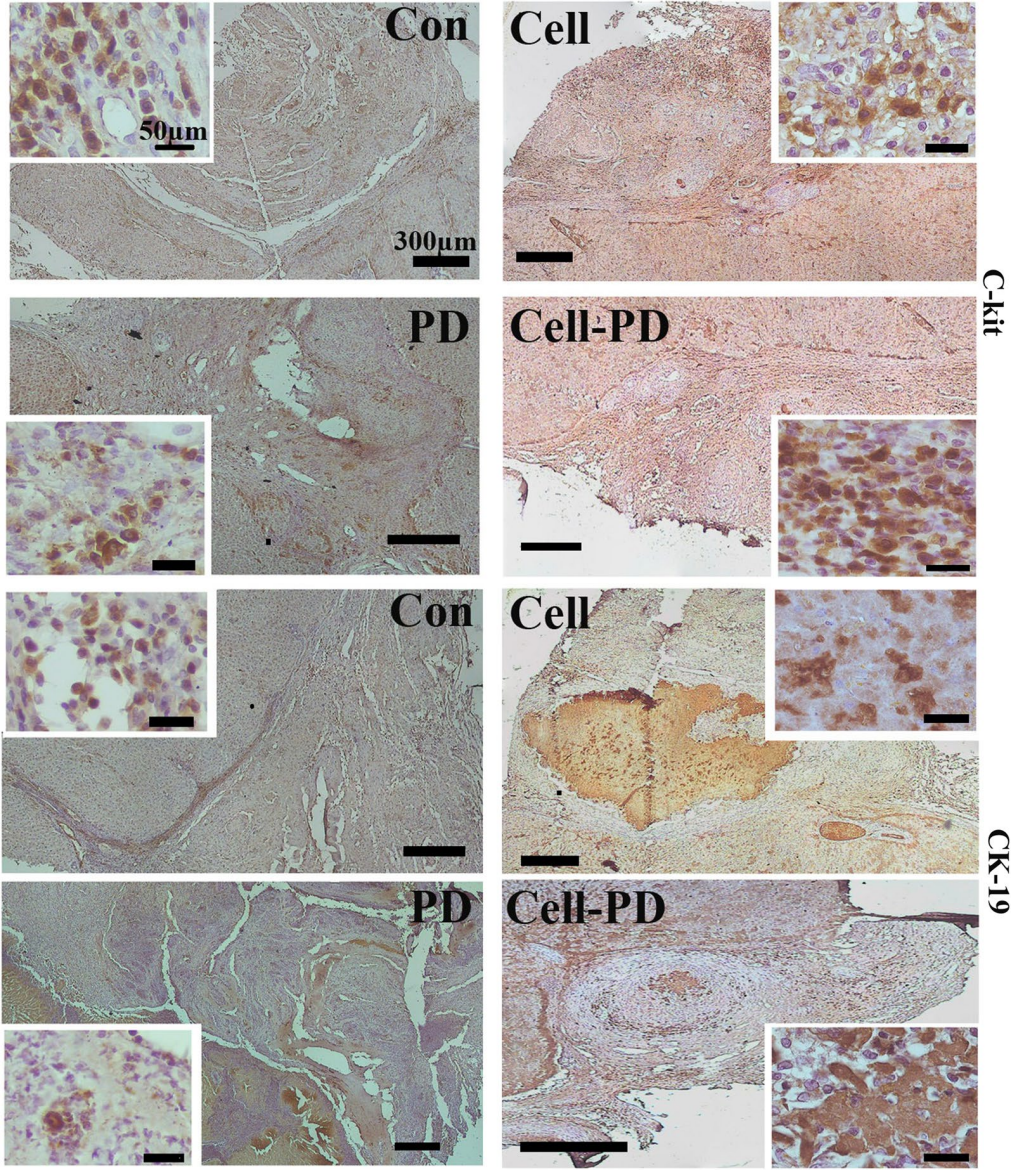
CK19-positive cells are representative of immature hepatoblasts and cholangiocytes migrating within the transplanted grafts after 2 weeks. In the cell and cell/prednisolone groups, the large aggregates of CK19-positive cells are present. Higher magnification (small square) showed that they had a hepatocyte phenotype. Extensive C-kit-labeled oval cell migration was detected by immunohistochemistry in all the grafts after 2 weeks. Higher magnifications were observed in small squares. Con: control

Immunohistochemical evaluations revealed that the hepatoblasts, cholangiocytes, oval cells, and hepatic stellate cells migrated or differentiated in the transplanted grafts. CK19-positive hepatoblasts and cholangiocytes scattered throughout the control and prednisolone-loaded scaffolds. In both cell-treated scaffolds, the immunoreaction to CK19 confirmed the presence of large aggregates of immature hepatocytes in the form of hepatic cords as well as scattered cells (Fig. 6). Some bile ducts also reacted with the CK19 antibody.

**Fig. 7:**
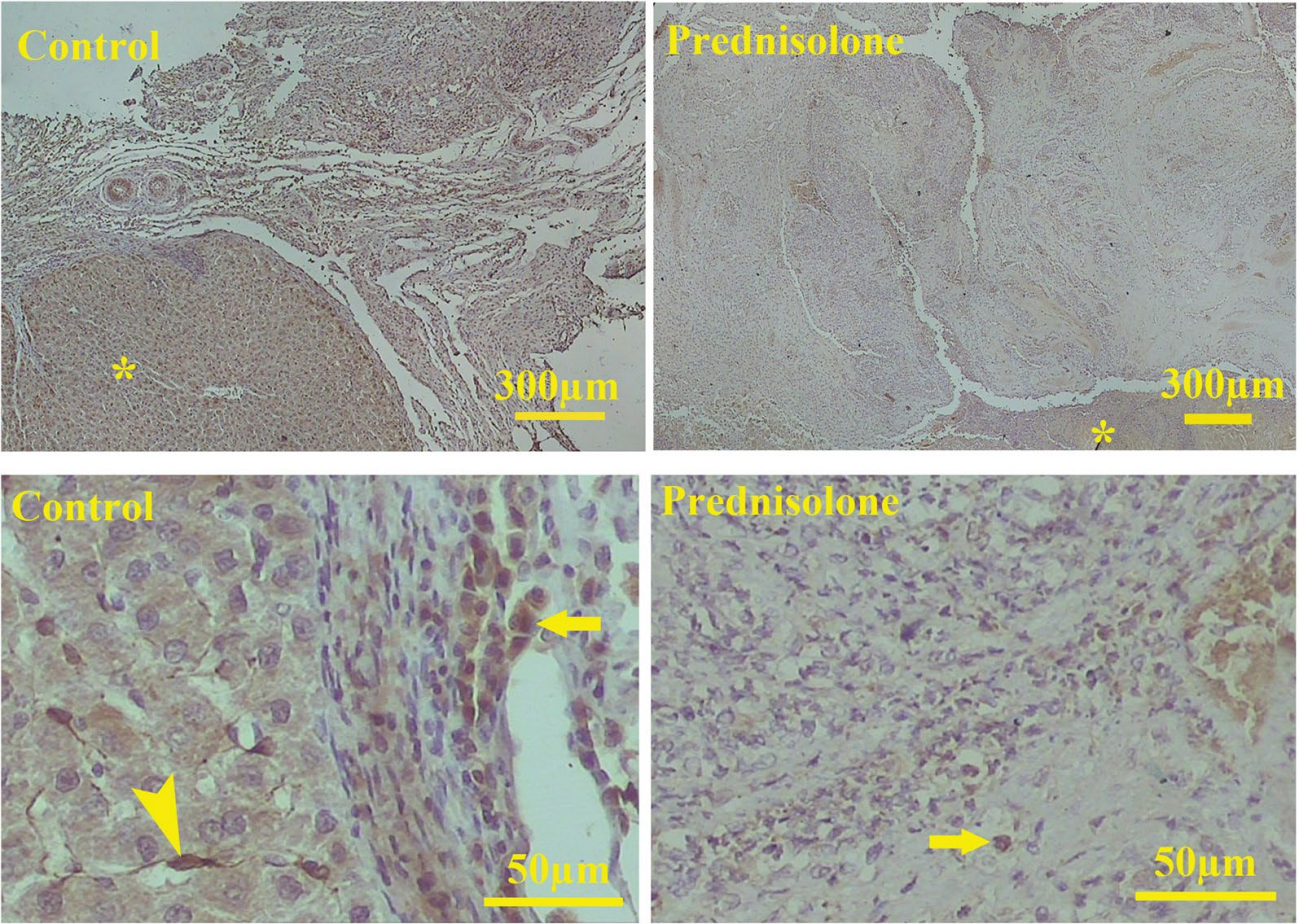
Stellate cell migration was detected by GFAP antibody. Microscopic examinations show that fewer stellate cells migrate to the prednisolone-loaded scaffold. Stellate cells in the intact part of the liver (arrowhead) and the graft (arrow). * indicates the intact part of the liver

GFAP antibody labeled the hepatic stellate cells that scattered in the regenerated as well as inflammatory regions (Fig. 6). The C-kit-positive oval cells also migrated into all the grafts and distributed throughout them (Fig. 7).

### Giant cells

**Fig. 8:**
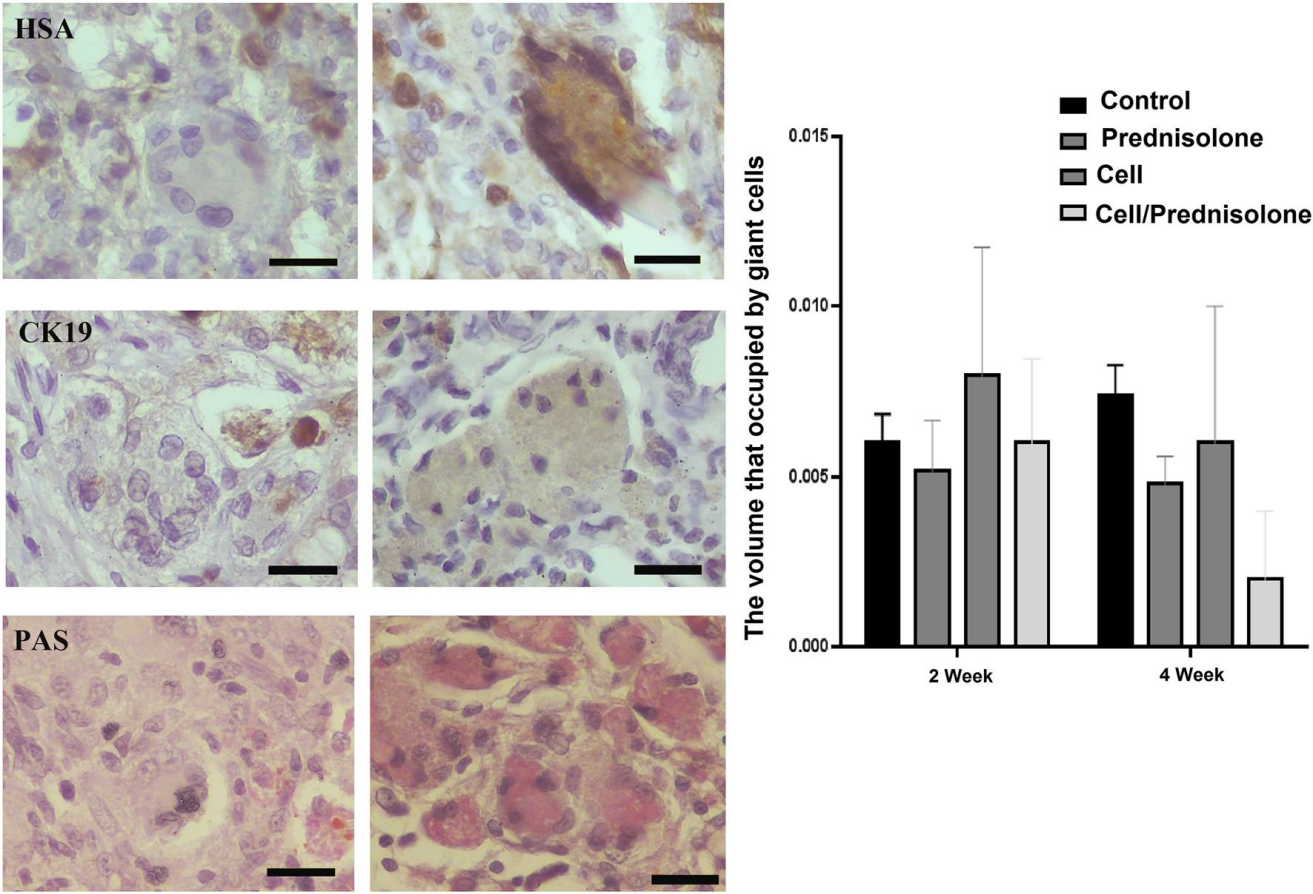
Foreign body giant cells. Hepatic giant cells reacted with CK19 and HSA antibodies as well as PAS staining. Multinuclear macrophages were HAS and CK19-negative and did not have glycogen in their cytoplasm. The graph shows that the volume of these cells was statistically the same in all groups. The scale bar is 50 µm

In all the scaffolds, foreign body giant cells with several nuclei were detected, and the quantitative data showed that the scaffold condition did not statistically impact the total giant cell volume. A few hepatic giant cells were stained with CK19, hepatocyte-specific antigen (HSA), and PAS that may be hepatic giant cells, and more abundant HSA and CK19-negative giant cells without glycogen in their cytoplasm were considered multinuclear macrophage (Fig. 8).

### Stereological estimations

**Fig. 9:**
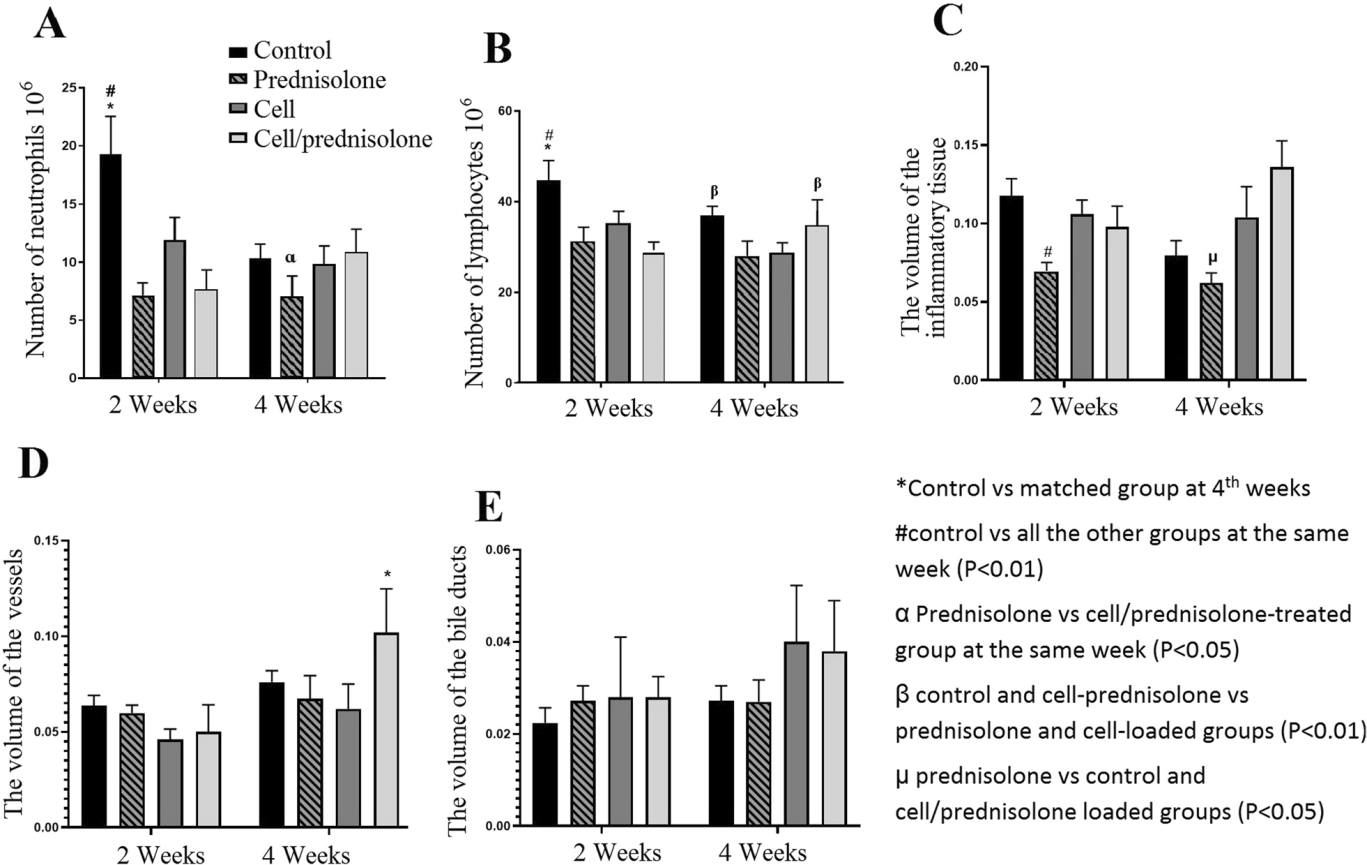
The number of the neutrophils (**A**), lymphocyte (**B**), and volume density of inflammatory tissue (**C**), vessels (**D**), and bile ducts (**E**). Prednisolone and MSCs accelerated the replacement of the neutrophil to the lymphocyte population as early as 2 weeks. Prednisolone and cell alleviated inflammation and immune cell migration. Angiogenesis was significantly higher in the cell/prednisolone-loaded group than the control (*P* = 0.0086), drug-loaded (*P* = 0.0004), and cell-loaded (*P* < 0.0001) groups at the 4th week after transplantation. A non-significant acceleration in the bile duct migration and formation was observed in both cell-loaded scaffolds compared to the control and prednisolone-loaded groups

The disector method revealed a significant decrease in the neutrophil count in the control as time progressed (*P* < 0.0001). All treated scaffolds significantly reduced the neutrophil count compared to the control at two weeks (*P* < 0.0001). However, at four weeks, the lowest number of the neutrophils was counted in the prednisolone-treated group, and it was significant compared to cell/prednisolone-loaded groups (*P* = 0.035, Fig. 9A). The number of the lymphocytes also decreased significantly in the control group as time progressed (*P* = 0.003). At two weeks, a lower number of the lymphocytes was counted in all the treated scaffolds than the control group (*P* < 0.0001). However, at four weeks, loading the scaffolds with either prednisolone or MSCs reduced the lymphocyte count compared to cell/prednisolone-loaded implants (*P* = 0.002, *P* = 0.004, respectively). The number of lymphocytes that invaded into the prednisolone- and cell-loaded implants remained statistically constant over time. This indicates that prednisolone and cell alleviated inflammation and immune cell migration (Fig. 9B).

The total volume of the inflammatory region was also estimated. In the control group, the data showed a significant decline in the volume of inflammatory tissues as time progressed (*P* = 0.002). The prednisolone-treated group contained a significantly lower inflammatory tissue volume than the controls at two (*P* = 0.004) and four weeks (*P* = 0.019). This indicates that prednisolone-loading in the engineered liver had an alleviatory effect on inflammation (Fig. 9C).

At 2 weeks, prednisolone and MSC loading had no negative impact on the volumetric density of the newly formed vessels, and the angiogenesis was statistically the same in all groups. As the time progressed, cell/prednisolone induced a significantly higher level of angiogenesis than the controls (*P* = 0.0086), drug-loaded (*P* = 0.0004), and cell-loaded (*P* < 0.0001) groups (Fig. 9D).

Volumetric analysis revealed that the bile duct formation was statistically the same in all the groups at both points of time (Fig. 9E).

**Fig. 10:**
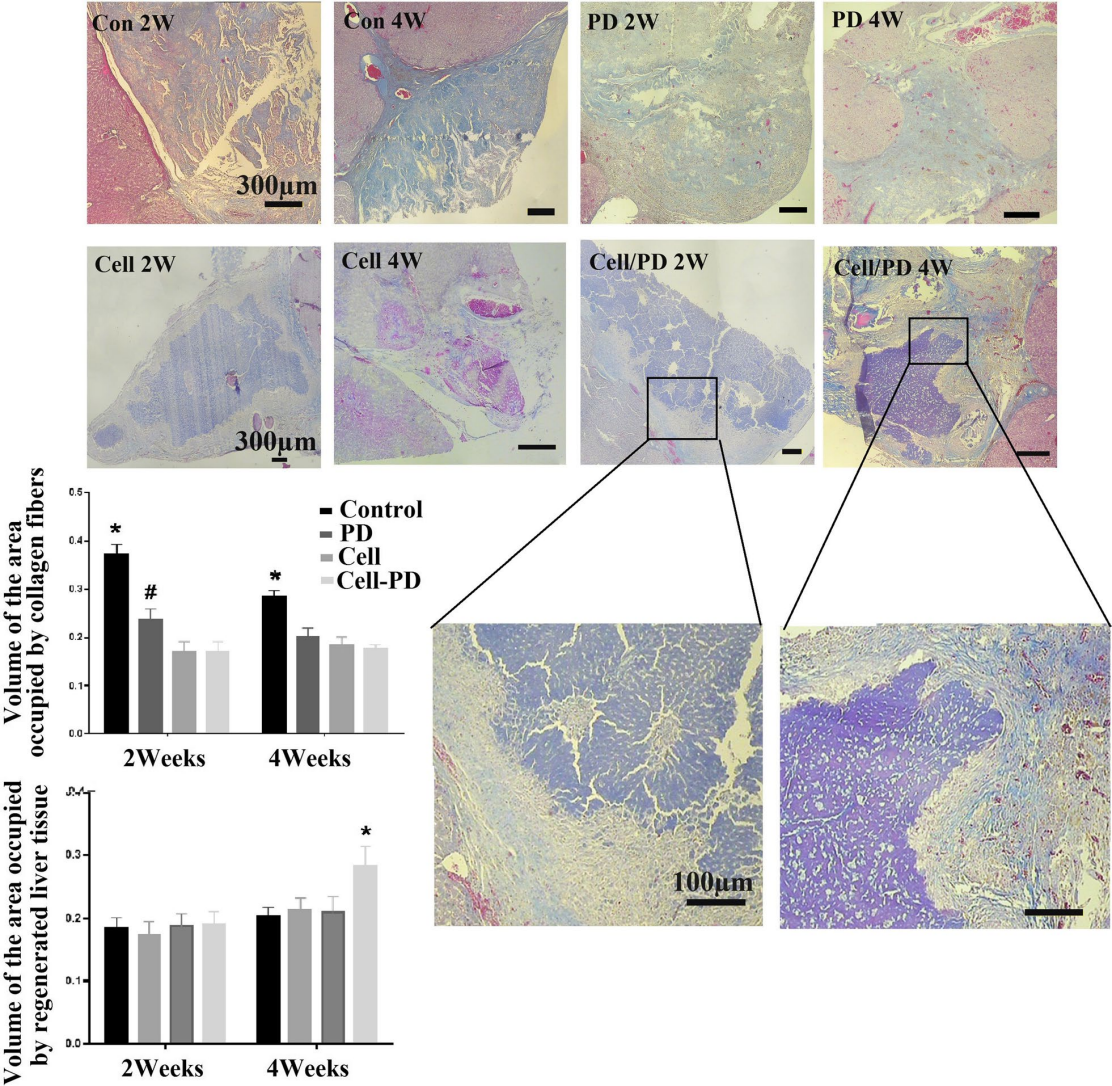
Trichrome Masson staining along with quantification of the volume occupied by fibrotic regions revealed the significant lower fibrogenesis in the grafts loaded with both cells and prednisolone. The volumetric density of the regenerated liver tissue was also significantly higher in the cell/prednisolone-treated group. PD, prednisolon. *Significant difference with the all other groups at same week (*P* < 0.05); ^#^Significant difference with the groups treated with cells and cells-PD at the same week (*P* < 0.05)

On the other hand, all the treatments led to a significant decrease in the volume of the collagenous tissue compared with the control group at 2 and 4 weeks. This indicates that the cell and prednisolone-loading positively influenced retaining the natural 3D ECM structure. Trichrome Masson staining also confirmed the volumetric analysis (Fig. 10). Treatment of the implants with MSCs was more efficient in decreasing fibrosis than prednisolone.

The volumetric density of the regenerated liver tissue was significantly boosted in the implants loaded with cell/prednisolone compared to the control (*P* < 0.0001), the MSCs (*P* < 0.0001), and the prednisolone-loaded group (*P* = 0.0001) after four weeks (Fig. 10).

## Discussion

The architecture of the decellularized livers has chemical and mechanical similarities to ECM in the naïve tissue. Extensive immune cell migration had detrimental impacts including fibrogenesis on the ECM architecture of the transplanted decellularized liver scaffolds [20] and it was restricted by loading the BM-MSCs and prednisolone. ECM provides chemical and mechanical signals that promote cell migration and differentiation and regulate cell and tissue phenotype [30]. According to two studies in 2019 by Naeem et al. and Shimoda et al., decellularized liver scaffolds can provide an appropriate environment for attracting hepatic specific cells and support cell proliferation [20, 31]. Naeem et al. also showed hepatoblast and oval cell migration into the transplanted decellularized scaffolds. However, in none of these studies, the migrating cells could reorganize properly to form the liver architecture. The current study also confirmed in vivo recellularization of decellularized scaffolds by all liver-specific cell types including the hepatoblasts, cholangiocytes, oval cells, stellate cells, and hepatic cord reorganization.

Naeem et al. also reported the presence of inflammatory cells in the transplanted decellularized scaffolds [22]. The presence of the neutrophils, lymphocytes, and M1 macrophages was reported to be involved in liver regeneration [32, 33]. These immune cells cooperate with the stellate cells to induce interstitial fibrotic tissue formation [34] leading to dramatic ECM modification. ECM modification and fibrosis may prevent liver reorganization despite extensive migration of liver-specific cells and bile duct formation. This evidence suggests that balancing the immune cell migration may inhibit the extensive fibrosis after decellularized scaffold transplantation [35]. In the present study, prednisolone, MSCs, or a combination of both were loaded on the liver scaffolds to alleviate the entrance of the inflammatory cells and reduce the formation of fibrous tissue.

To balance the immune cell infiltration, we loaded the transplanted decellularized scaffolds with prednisolone; the data showed a significant reduction in the neutrophil and lymphocyte count, and collagen fiber formation compared to the controls. Although the neutrophils help the liver repair, they can cause cell damage by producing proteases and reactive oxygen species [36]. In these conditions, immunosuppressive drugs, including glucocorticoids, are used to limit inflammation. Systematic administration of prednisolone can improve the biomechanical and histological abnormalities caused by liver diseases [37]. However, glucocorticoids are not inherently toxic; long-term or high dose consumption cause liver failure and increases liver enzymes [38]. To limit these side effects, we loaded prednisolone locally in the decellularized liver and found it could alleviate inflammation and fibrosis.

Although neither prednisolone nor BM-MSC loading had a beneficial or detrimental impact on the hepatocyte migration and differentiation, the combination of both led to a significant increase in the volume of the regenerative liver tissue. There are contradictory data regarding the impact of synthetic glucocorticoids such as prednisolone on liver structure and functions. While a human retrospective cohort study indicated prednisolone is safe [39], animal studies indicated some adverse effects such as glycogen depletion [40]. Evidence indicates a correlation between the hepatocyte size and glycogen content in normal rat liver [41]. Glucocorticoids such as prednisolone have dual effects on the hepatocytes. For instance, the oral administration of glucocorticoids led to glycogen depletion by either autophagy or inhibition of glycogen synthase. On the other hand, overexpression of genes involved in the gluconeogenic pathway and glycogen synthesis has been reported in domestic animals after dexamethasone administration [42]. Besides, hepatocyte enlargement along with a decrease in hepatocyte proliferation were recorded after glucocorticoid administration [43, 44]. Therefore, prednisolone loading did not change the liver tissues' total volume, because the decrease in hepatocyte division might be compensated by an increase in the cell size. However, the effect of these glucocorticoids are dose-dependent and it has been recorded that it is safe in therapeutic doses [45]. The impact of MSC infusion in liver regeneration is also contradictory. While reports on partial hepatectomized rats revealed both hepatocyte proliferation and enlargement after general injection of MSCs [46], MSC infusion into the porcine portal vein did not significantly impact on the hepatocyte size and proliferation [47]; this is in the same line with our data. However, MSC treatment could accelerate liver regeneration [47]. We also found hepatocyte organization improvment in both MSC-treated scaffolds. These controversies may be attributed to the different species and the way of introducing MSCs.

The injection of the hepatic growth factor-overexpressed BM-MSCs ameliorated liver functions in cirrhosis model rats [48]. MSCs exposed to synthetic glucocorticoids such as dexamethasone can express a higher level of hepatic growth factor [49]. This evidence may explain the synergic impact of prednisolone and BM-MSC in the reorganizing of the migrating hepatic-specific cells in the decellularized scaffold.

Mesenchymal stem cells have not only multiple differentiation potential but also the ability to modulate the host immune system. Hence, they are an ideal source for utilization in reconstructive medicine [50]. We found that in the short time, loading of the decellularized scaffold with BM-MSCs or BM-MSC/prednisolone significantly reduced the number of the neutrophil and lymphocytes, but not the volume occupied by inflammatory cells, probably due to reduction in the immune cell density or increase in the number of other immune cell types in these groups. After four weeks, the volume of the inflammation area increased in the group treated with BM-MSCs/prednisolone. In vitro administration of synthetic glucocorticoids such as dexamethasone has been reported to reduce the immunomodulatory activity of MSCs [49]. Injecting the dexamethasone with MSCs to the mouse liver fibrosis model was found to interfere with the anti-inflammatory effects of MSCs and revoke their anti-fibrotic effects [51]. Our data also indicated that such a combination reduced the anti-inflammatory property, but not the anti-fibrotic effects of the MSCs.

Loading of prednisolone, BM-MSC, and their combination diminished liver fibrosis. However, both cell-containing scaffolds were superior to the prednisolone-treated one in reducing the collagen fiber deposition. Prednisolone has been reported to suppress the collagen synthesis by the hepatocytes [52]. Intraportal vein infusion or direct injection of MSCs to the liver site has been reported as a therapeutic strategy to reverse liver fibrosis [53], which is in line with our data. We could trace the PKH-labeled MSCs for up to two weeks in the scaffold. Therefore, they could be alive to produce cytokines which are critical for reversing fibrogenesis [53]. On the other hand, it has been shown that glucocorticoids limit hepatic stellate cell migration [54]. Although we did not quantify the number of migrating stellate cells, microscopic examinations revealed that a lower number of stellate cell migrated into the scaffolds loaded by prednisolone compared to the control scaffolds. The prolonged activity of these cells can lead to the formation of fibrous tissue [55]. Besides, in vivo glucocorticoid administration to animal models reduced liver fibrosis and the expression of the genes involved in fibrosis [56].

In the chronic inflammatory stage, the foreign body giant cells invade into the biological scaffolds such as implanted decellularized tissues [57]. Our observations indicate the presence of two types of giant cells in all the grafts. A small subpopulation of giant cells reacted with PAS, CK19, and HSA, while most of them were negative for such markers. In a few pathological cases such as autoimmune disorders, viral infections, drug reactions, and congenital metabolic diseases, multinucleated hepatic cells are formed due to multiple divisions of the hepatocyte nucleus without dividing the cytoplasm [58]. The giant cells that reacted with the hepatocyte markers might be hepatic giant cells. PAS staining showed that they were functional as well. HAS- and CK19-negative giant cells may represent the fused macrophages involved in immunoreaction to the foreign scaffold.

Vasculogenesis of the engineered grafts is a critical point in the regeneration of the impaired tissues. MSC therapy has been shown to improve vasculogenesis through the production of proangiogenic and proarteriogenic factors [59]. Although a previous study reported that the recellularization of natural rat liver scaffolds induced differentiation of loaded-MSCs into endothelial linage, our stereological analyses revealed that neither MSC nor prednisolone loading had a beneficial impact on angiogenesis in the liver decellularized scaffold. However, the combination of both significantly accelerates vasculogenesis in short term. In the same line with our data, an in vitro study reported corticosteroids boosted the angiogenic properties of MSCs, while these cells still alleviated the immunomodulatory activity. Besides, a low dose of dexamethasone led to an increase in vascular endothelial growth factor expression by MSCs, and pre-treatment of MSCs with dexamethasone was recommended for reinforcing their angiogenic properties [49]. Vascular endothelial growth factor produced by MSCs is an essential factor in liver morphogenesis, and its presence leads to the formation of sinusoids [60].

Although transplantation of the decellularized scaffolds showed that prednisolone loading had no detrimental impact on the hepatocyte migration and bile duct formation, in vitro exposure of the HepG2 cell line with the drug revealed some extent of cytotoxicity. On the first day of cytotoxicity assay, when the concentration of releasing prednisolone from decellularized scaffolds was high, the number of viable hepatocytes was significantly less than the controls. The test revealed that the drug was released in the first 5 h of incubation. We also compared the cytotoxicity of the prednisolone loaded in a thin alginate film to provide a 2D environment. While the cell viability in the presence of prednisolone and 2D condition was similar to control culture, it seems the releasing pattern of prednisolone is different in the alginate and decellularized scaffold. Prednisolone has also an anti-tumor effect, diminishes cell proliferation, and induces apoptosis in liver cancer cells such as HepG2 [61].

## Conclusion

The results indicated that the grafts provided an appropriate microenvironment for liver-specific cell as well as inflammatory cell migration. The grafts could be repopulated with the cells such as the hepatoblasts, stellate cells, oval cells, fibroblasts, cholangiocytes, lymphocytes, giant cells, and neutrophils. Bile ducts can also be formed in different parts of the grafts. Loading of prednisolone in decellularized liver scaffolds reduced inflammation and prevented graft fibrosis. Also, recellularization of bioscaffolds by BM-MSCs caused liver reorganization and formation of the construct similar to the portal spaces and hepatic cords around the sinusoids. The concurrent loading of prednisolone and BM-MSCs on the liver scaffolds could not prevent inflammation due to the counteraction of glucocorticoids with the anti-inflammatory effects of MSCs, however, it could show a synergistic impact on diminishing fibrosis, increasing angiogenesis and the volume of the repaired area, and liver reorganization.

## Supplementary Information

**Additional file 1: Fig. S1.** The flow chart shows the in vivo treating methods.

**Additional file 2: Fig. S2.** The flow cytometry indicated the MSCs isolated from bone marrow expressed CD90 (99.8%) and CD44 (100%). The cells were negative for DC34 and CD45 (A). They also showed pluripotency as indicated by the capability to differentiate into the osteoblasts (B) and adipocytes (C).

## Data Availability

All data generated or analyzed during this study are included in this published article.

## Abbreviations

ANOVA: Analysis of variance
BM-MSC: Bone marrow-derived mesenchymal stem cells
CK19: Cytokeratin 19
ECM: Extracellular matrix
FBS: Fetal Bovine Serum
GFAP: Glial fibrillary acidic protein
H&E: Hematoxylin and eosin
HPLC: High-performance liquid chromatography
HSA: Hepatocyte specific antigen
LG-DMEM: Low-glucose Dulbecco's modified Eagle's medium
MMPs: Matrix metalloproteinases
MSCs: Mesenchymal stem cells
MTT: 4,5-Dimethyl thiazolyl-2)-2,5-diphenyltetrazolium bromide
OD: Optical density
PAS: Periodic acid Schiff
PBS: Phosphate-buffered saline
ROS: Reactive oxygen species
SEM: Scanning electron microscopy
SLES: Sodium Lauryl Ether Sulfate
