# Supplementary figures and images for: Prednisolone and mesenchymal stem cell preloading protect liver cell migration and mitigate extracellular matrix modification in transplanted decellularized rat liver
Source: Stem Cell Res Ther. 2022 Jan 28;13:36. doi: 10.1186/s13287-022-02711-8 (PMC8800282; doi:10.1186/s13287-022-02711-8)

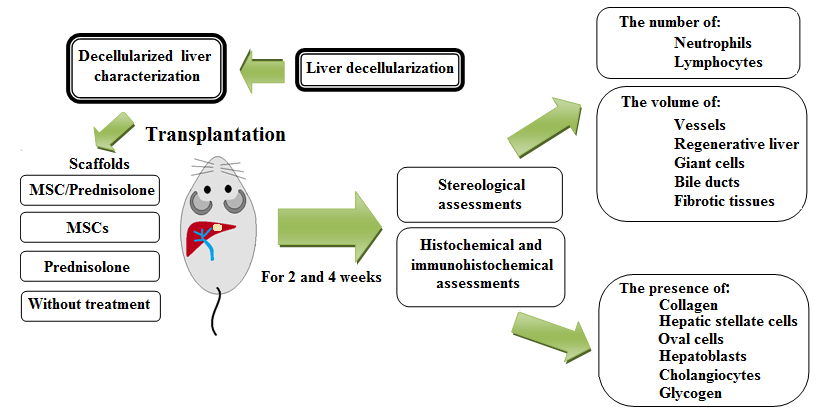

Supplement: Supplementary file 1 — Additional file 1: Fig. S1. The flow chart shows the in vivo treating methods. [file 13287_2022_2711_MOESM1_ESM.png]

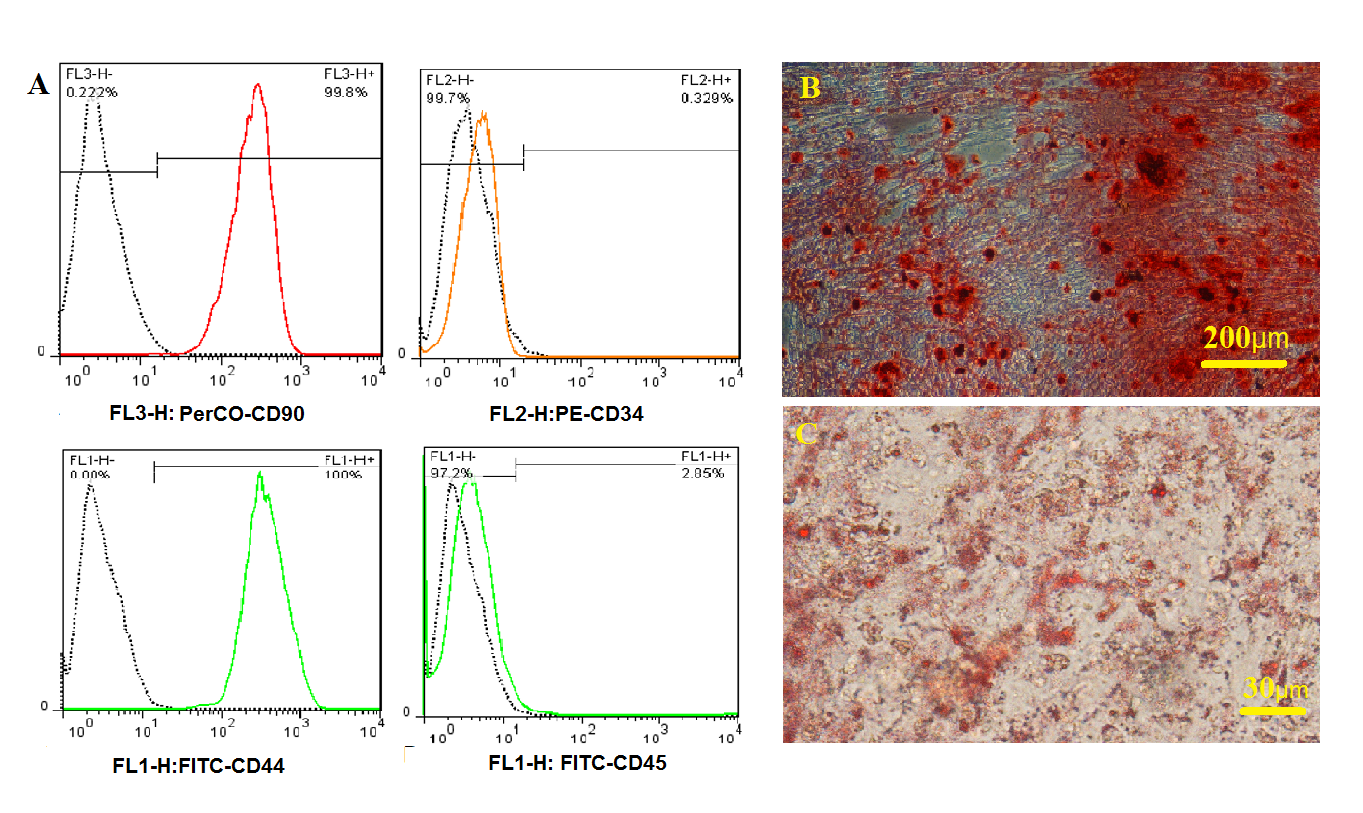

Supplement: Supplementary file 2 — Additional file 2: Fig. S2. The flow cytometry indicated the MSCs isolated from bone marrow expressed CD90 (99.8%) and CD44 (100%). The cells were negative for DC34 and CD45 (A). They also showed pluripotency as indicated by the capability to differentiate into the osteoblasts (B) and adipocytes (C). [file 13287_2022_2711_MOESM2_ESM.png]
